# Intrinsic refractive index matched 3D dSTORM with two objectives: Comparison of detection techniques

**DOI:** 10.1038/s41598-018-31595-z

**Published:** 2018-09-06

**Authors:** Nora C. Schmidt, Martin Kahms, Jana Hüve, Jürgen Klingauf

**Affiliations:** 1 Fluorescence Microscopy Facility Münster (FM)2, Institute of Medical Physics and Biophysics, Center For NanoTechnology (CeNTech), Heisenbergstraße 11, 48149 Münster, Germany; 2 Department of Cellular Biophysics, Institute of Medical Physics and Biophysics, University of Münster, Robert-Koch-Straße 31, 48149 Münster, Germany; 3 IZKF Münster and Cluster of Excellence EXC 1003, Cells in Motion, CiM, 48149 Münster, Germany

## Abstract

We have built a setup for 3D single molecule localisation microscopy (SMLM) where a very high resolution is achieved by, firstly, the use of two objectives instead of one and, secondly, minimizing optical aberrations by refractive index matching with a glycerol-water mixture as immersion medium in conjunction with glycerol-immersion objectives. Multiple optical paths of the microscope allow to switch between astigmatic and interferometric localisation along the optical axis, thus enabling a direct comparison of the performance of these localisation methods.

## Introduction

Single molecule localisation microscopy techniques as stochastic optical reconstruction microscopy (STORM)^1^, direct stochastic optical reconstruction microscopy (dSTORM)^2^, photoactivated localisation microscopy (PALM)^3^, fluorescence photoactivation localisation microscopy (FPALM)^4^ and ground state depletion followed by individual molecule return (GSDIM)^5^ elegantly circumvent the diffraction limit of optical microscopy by imaging single fluorescent molecules which stochastically switch between a fluorescent and a non-fluorescent state. Spatial separation of the fluorescent emitters allows to obtain the dye molecule coordinates, and therefore to resolve the stained structures, with a precision at least ten-fold better than the optical resolution. Originally, this approach was limited to 2D imaging, but subsequently various techniques have been developed to also obtain the axial (*z*) coordinate and reconstruct 3D images. They all involve some means to make the single molecule images more *z*-dependent, for example by inserting a cylindrical lens into the detection beam path which adds astigmatic distortion to the images^6^, imaging two distinct axial planes simultaneously^7^ or by capturing the fluorescent light with two objectives and bringing it to interference^8–10^, which causes *z*-dependent intensity variations. The localisation precisions achievable with these methods were investigated theoretically^11^ and a direct experimental comparison of biplane and astigmatic detection was performed for a microscope with a single water-immersion objective^12^, but not for two-objective schemes such as interferometric detection.

The localisation precision surpasses the resolution limit, but nevertheless depends on the size of the microscope’s point spread function (PSF) and therefore its numerical aperture (NA). However, objective lenses with high NA which are required for good optical resolution mostly use oil as immersion medium due to the resulting high NA, whereas biological samples usually have to be imaged in aqueous buffers. This leads to a refractive index mismatch between cover glass and watery sample, which causes both aberrations and loss of signal^13^. For single molecule localisation, this is unfavourable as both effects degrade the localisation precision. Furthermore, wavefront deformations will distort the interference pattern when interferometric detection is used.

One approach to circumvent the degrading effects of refractive index matching is to include a wavefront correction mechanism into the system. This was successfully implemented for astigmatic detection^14^ and interferometric detection^15^. However, a more straightforward approach is to prevent the refractive index mismatch and the associated degrading effects by matching the refractive indices of immersion medium, cover glass and sample. For example, when water-immersion objectives were employed for 3D STORM with astigmatic detection^16^, they yielded better results than oil-immersion objectives. But due to the lower refractive index of water, water-immersion objectives have a lower NA (typically 1.2), which increases the size of the PSF and reduces the number of collected photons, effects that are disadvantageous for the localisation precision. For oil-immersion objectives with their high NA of 1.4 or more, a refractive index match may be achieved by embedding the sample in 2,2’-thiodiethanol (TDE) instead of aqueous buffers^17,18^. But this method has the disadvantage that for an exact refractive index matching, the aqueous medium has to be replaced stepwise with TDE to avoid artefacts due to osmosis. Glycerol-immersion objectives may be another alternative. With NA = 1.35, their numerical aperture is higher than those of water-immersion objectives, but not as high as with oil immersion. Their advantage is that embedding biological samples in glycerol-containing media is easier feasible than in TDE. No stepwise procedure is required, it is very inert, and many mounting media for fluorescence microscopy contain glycerol anyway. The required refractive index for glycerol immersion objectives is slightly higher than for silicone oil immersion objectives, which have recently been used to enable refractive index matching for live cell confocal spinning disc microscopy^19^.

Here we have investigated the performance of glycerol immersion objectives^20^ for 3D localisation microscopy with astigmatic and interferometric detection. These objectives were already successfully employed for superresolution microscopy^21^, but as far as we know they have not yet been used for (d)STORM. Testing different detection methods on the same setup with identical optics enables a direct comparison of their localisation precision under these conditions.

Interferometric detection requires a setup with two objectives. For astigmatic detection, this is advantageous as well^22,23^, because twice as many photons are detected, which increases the localisation precision by $\sqrt{2}$. For such a scheme with two objectives, refractive index matching is especially beneficial. With a mismatch between cover glass and sample buffer, aberrations are more prominent deep inside the sample. Accordingly, in a microscope with two objectives the localisation precision would be degraded for one of the objectives because there is a layer of several micrometers of buffer between sample and objective. However when the refractive index of the buffer is adjusted, the thickness of the buffer film has no impact on the image quality.

## Results

### Degradation of localisation precision due to refractive index mismatch

To demonstrate the effect of refractive index mismatch, we performed simulations of the detection PSF generated by an oil immersion objective. As detection PSF model, we employed the distribution of the electromagnetic field generated by a radiating dipole at the focus of a microscope with infinity-corrected objectives^24^, which treats the electromagnetic fields as vectors, not as scalar quantities.

**Figure 1:**
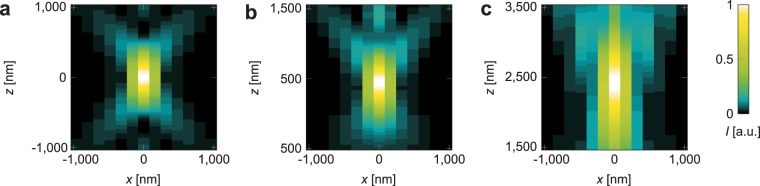
Impact of refractive index mismatch on the PSF, calculated for an oil immersion objective with half-aperture angle *α* = 60°, focusing into an aqueous sample. (**a**) No refractive index mismatch (*n*_*s*_ = *n*_1_ = 1.52). (**b**) Refractive index mismatch (*n*_*s*_ = 1.33, *n*_1_ = 1.52), depth in sample *d*_*s*_ = 1 *μ*m. (**c**) Refractive index mismatch (*n*_*s*_ = 1.33, *n*_1_ = 1.52), depth in sample *d*_*s*_ = 10 *μ*m. Note that the focus is shifted substantially due to the path length difference.

A refractive index mismatch between cover glass and sample causes an optical path length offset *δ* which depends on the refractive indices of cover glass and immersion medium *n*_1_, on the refractive index of the sample *n*_*s*_ and on the distance between focus and cover glass *d*_*s*_^13^,

1 $$\delta ={d}_{s}[{n}_{1}\,\cos \,({\theta }_{1})-{n}_{s}\,\cos \,({\theta }_{s})]$$

where *θ*_1_ and *θ*_*s*_ are the polar angles in the immersion medium and the sample, respectively. This path length difference leads to a phase offset which has to be included into the calculation of the electromagnetic fields. The deflection of the electromagnetic field vectors due to refraction at the glass-sample interface was neglected, as well as the loss of intensity. For a more accurate investigation of the impact of refractive index mismatch on localisation precision, these effects would have to be included; the present example only serves to estimate the influence of the additional optical path length. The resulting distributions of the intensity *I* without aberration and with *d*_*s*_ = 1 *μ*m and *d*_*s*_ = 10 *μ*m are shown in Fig. 1. They were calculated for an oil-immersion objective with *n*_1_ = 1.52 and an aqueous sample with *n*_*s*_ = 1.33. The half-aperture angle *α* was set to *α* = 60°, corresponding to NA = 1.32, because larger angles would require taking into account the deflection of the field vectors.

From these intensity distributions, we calculated the theoretical limits of the lateral (*xy*) localisation precision according to^25^,

2 $${u}_{a}={\{N{\int }_{-{\rm{\infty }}}^{{\rm{\infty }}}{\int }_{-{\rm{\infty }}}^{{\rm{\infty }}}\frac{1}{\mu (x,y;a)}{[\frac{{\rm{\partial }}\mu (x,y;a)}{{\rm{\partial }}a}]}^{2}{\rm{d}}x{\rm{d}}y\}}^{-1/2}$$

where *a* is the quantity to be estimated from the measurement (in this case, the fluorescent molecule’s *x* position), *N* the number of detected photons and *μ* the intensity distribution according to the PSF model normalized so that ${\int }_{-\infty }^{\infty }{\int }_{-\infty }^{\infty }\,\mu (x,\,y;\,a)\,{\rm{d}}x{\rm{d}}y=1$. All computations were carried out in MATLAB® (The MathWorks, Inc., Natick, Massachusetts, USA). For a fluorescent Alexa568 molecule emitting *N* = 2826 photons^26^, with a hypothetical background of *N*/1000 photons per camera pixel of size 160 nm × 160 nm in the object space, we found that the mean of *u*_*x*_ on an axial range of 0.5* μ*m around the focus was *u*_*x*_ = 3.1 nm without mismatch (*n*_*s*_ = *n*_1_ = 1.52). When a refractive index mismatch was assumed (*n*_*s*_ = 1.33, *n*_1_ = 1.52), the limit of localisation precision was *u*_*x*_ = 3.5 nm at a depth of *d*_*s*_ = 1 *μ*m in the sample and *u*_*x*_ = 4.2 nm at *d*_*s*_ = 10 *μ*m depth. Accordingly, in this (somewhat simplified) model the localisation precision is degraded due to the mismatch by 13% and 37%, respectively.

This degradation can be avoided by matching the sample buffer’s refractive index to that of cover glass and immersion medium, as was done in this work. For this, we used glycerol immersion objectives and fused silica cover glasses with *n* = 1.46 instead of oil immersion objectives and standard cover glasses with *n* = 1.52. These have an half-aperture angle of *α* = 68.5°, which is close to the half-aperture angle *α* = 67.3° of typical oil immersion objectives with NA = 1.40. Accordingly, the numbers of detected photons should be similar for both types of objectives as they cover nearly the same solid angle.

A more detailed discussion of the impact of refractive index mismatch and NA on the localisation precision and on the limitations of our model can be found in the Supplementary Information, as well as additional simulations.

However, with the index matching as described here, a residual refractive index mismatch may still arise between buffer and the cytosol or the nucleus of cells when biological samples are imaged. Accordingly, the approach presented here cannot completely prevent aberrations arising from refractive index inhomogeneities, it merely eliminates a major source of such aberrations.

### Comparison of localisation precisions

To compare the performance of astigmatic and interferometric detection, localisation precisions for the *x*, *y* (lateral plane) and *z* (optical axis) direction were estimated for various *z* positions by imaging fluorescent beads.

To measure the localisation precision, a fluorescent bead was moved along the optical axis and at each *z* position, 20 images were taken. For each *z* step, the *x*, *y* and *z* positions of the bead and their standard deviations were estimated. Results for *z* steps where the bead had been successfully localised less than ten times were discarded in order to obtain the standard deviation from a sample of sufficient size. When these beads were illuminated with the 561 nm laser, with the respective AOTF transmission set to very low values of about 1.2%, approximately 17000 photons per frame were detected, with a mean background of 1.4 photons per pixel. The electron multiplying process in the EMCCDs leads to an increase in noise of about $\sqrt{2}$ which corresponds to cutting the photon number in half^27^. This effect was included in these calculations.

The relative error of the standard deviation is assumed as 1/(2 × 10)^1/2^ ≈ 0.2, the expected uncertainty of the standard deviation for a sample of ten values. As described above, the sample size may range between ten and twenty, but here an upper bound for the error was estimated.

The experimental results were compared to the theoretical limits of localisation precision *u*_*x*,*y*,*z*_ according to Eq. (2).

When astigmatic detection is employed, the cylindrical lens causes an offset of the focal length between the *x* and *y* direction. For our system where the focal length of the cylindrical lenses is 300 mm and their distance to the tube lenses 160 mm, this offset is about 700 nm in the sample space, as may be derived with geometrical optics. This offset causes an asymmetric, quadratic wavefront aberration^11,28^ which has to be included into the calculation of the electrical fields arriving at the detectors.

For interferometric detection, the total electrical field arriving at the cameras was computed as the sum of the fields from each interferometer arm with their respective phases^8,9,11^.

**Figure 2:**
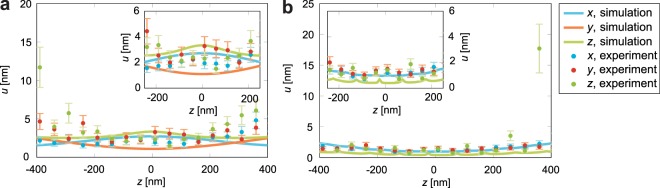
Localisation precisions *u* against axial position *z* with TransFluoSpheres® for *x*, *y* and *z*. (**a**) Astigmatic detection. (**b**) Interferometric detection. Insets show magnifications of half a micrometer around the focus. For interferometric detection, simulated localisation precisions are identical for *x* and *y*.

The measured localisation precisions are shown in Fig. 2, as well as the theoretical limits of localisation precision obtained from simulations of the PSF. The focus position is at *z* = 0.

When astigmatic detection was employed, localisation precisions *u*_*x*_ = (2.0 ± 0.1) nm, *u*_*y*_ = (2.8 ± 0.2) nm and *u*_*z*_ = (2.6 ± 0.2) nm were measured for *z* ∈ (−250 nm, 250 nm). Here the theoretical limits were *u*_*x*_ = 2.5 nm, *u*_*y*_ = 1.3 nm and *u*_*z*_ = 2.9 nm. Due to the astigmatic aberration, the *x* and *y* localisation precisions are slightly degraded and the value for *y* lies above the theoretical limit, but on the other hand the localisation precision is nearly isotropic.

In contrast to interferometric detection, the particle positions are determined independently from the data of both cameras when astigmatic detection is used. This allows to compare the localisation precisions of the two-objective-scheme to the case where the images are obtained by only one objective and detector. For classic astigmatic localisation with a single objective lens, the measured localisation precisions were *u*_*x*_ = (2.6 ± 0.1) nm, *u*_*y*_ = (3.7 ± 0.2) nm and *u*_*z*_ = (3.7 ± 0.2) nm. Accordingly, the second objective enhanced the localisation precision by a factor of 1.3, 1.3 and 1.5, respectively. The mean enhancement is 1.4 which agrees well with the predicted value $\sqrt{2}$.

The observed values for interferometric detection were *u*_*x*_ = (1.2 ± 0.1) nm, *u*_*y*_ = (1.4 ± 0.1) nm and *u*_*z*_ = (1.2 ± 0.1) nm. The precisions lie slightly above the theoretical limits *u*_*x*_ = 1.1 nm, *u*_*y*_ = 1.1 nm and *u*_*z*_ = 0.5 nm, but reach lower values than astigmatic detection. The deviation of *u*_*z*_ from the theoretical value seems to be mainly caused by a reduced modulation depth of the interference pattern. Loss of modulation depth is partly induced by polarization effects, which can be avoided using a polarizing beamsplitter^9^, at the cost of necessitating four detection channels instead of three and an additional Babinet-Soleil compensator. Another contribution is reduced coherence of the detected light. Due to its spectral width and low coherence length, coherent detection of fluorescent light turned out to be a major challenge, which makes this detection method susceptible to imperfect adjustment and flaws in optical components, rendering it the technically most demanding 3D localisation modality.

It is noteworthy that interferometric detection reaches better results for all three dimensions. In astigmatic detection, the astigmatic aberration caused by the cylindrical lens slightly degrades the *x* and *y* localisation precision. In our microscope, we employed a cylindrical lens with a short focal length in order to obtain a strong astigmatism, enabling good *z* localisation precision at the cost of *x* and *y* localisation precision. In contrast, the *x* and *y* localisation precision is not compromised in interferometric detection.

To compare the 3D resolving power of these two detection schemes, we calculated a localisation volume *V* with $V=\frac{4\pi }{3}{u}_{x}{u}_{y}{u}_{z}$, the volume of a rotational ellipsoid^29^, where the semi-axes *u*_*x*,*y*,*z*_ are the mean values for *z* ∈ (−250 nm, 250 nm). The resulting volumes were (60 ± 10) nm^3^ for astigmatic detection and (8 ± 2) nm^3^ for interferometric detection. This underlines that interferometric detection reaches by far the best 3D localisation precision.

A measurement with less bright emitters, which emit photon numbers comparable to bright dSTORM dyes, can be found in the supporting information.

### Resolving power for stained microtubules

To investigate the performance of the setup in dSTORM experiments, microtubules of 3T3 cells were stained with two different commonly used dSTORM dyes, Alexa Fluor 647 (Alexa647) for the red spectral range and Alexa Fluor 568 (Alexa568) for the orange spectral range. As sub-resolution structures of homogeneous, well-known size, microtubules are ideally suited to test superresolution microscopy setups^6^.

**Figure 3:**
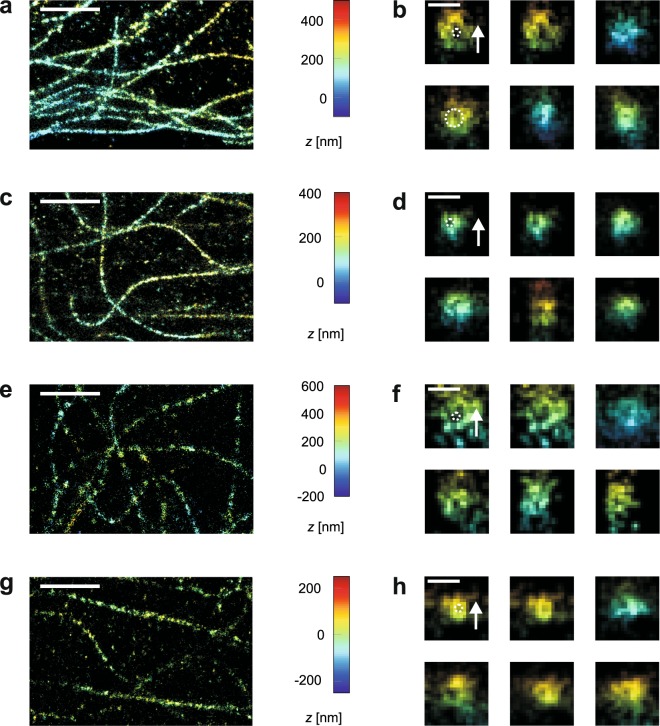
dSTORM images of stained microtubules. (**a**) Overview image of Alexa647 staining for astigmatic detection, scale bar 2 *μ*m. (**b**) Five axial slices through microtubules stained with Alexa647 for astigmatic detection, scale bar 100 nm. (**c**) Overview image of Alexa647 staining for interferometric detection, scale bar 2 *μ*m. (**d**) Five axial slices through microtubules stained with Alexa647 for interferometric detection, scale bar 100 nm. (**e**) Overview image of Alexa568 staining for astigmatic detection, scale bar 2 *μ*m. (**f**) Five axial slices through microtubules stained with Alexa568 for astigmatic detection, scale bar 100 nm. (**g**) Overview image of Alexa568 staining for interferometric detection, scale bar 2 *μ*m. (**h**) Five axial slices through microtubules stained with Alexa568 for interferometric detection, scale bar 100 nm. For each method, the first axial slice is also shown with a dashed ring of diameter 25 nm, indicating the microtubule thickness, and a white arrow denoting the optical axis.

The glycerol which was added to the dSTORM imaging buffer in order to enable refractive index matching did not markedly alter the number of detected photons and the time the molecules spent in the fluorescing state (see supporting information), therefore it was possible to reconstruct superresolved 3D images of microtubules for astigmatic and interferometric detection (Fig. 3). Partly, the microtubules appear not homogeneously stained but dotted. Comparison with 2D STED images (see Supplementary Information) of similarly stained samples showed discontinuous structures as well, which indicates that this is mainly not an artefact of single molecule localisation but a result of rather sparse staining; the distance between dye molecules is larger than the localisation precision. Of course, insufficient detection of dye molecules during dSTORM imaging and data analysis may further contribute to a sparse number of localisations.

Axial reconstructions of the microtubules were created by projecting localisations onto a plane perpendicular to the microtubule (Fig. 3); the mean depth of these projections was about 200 nm. In these axial slices, astigmatic as well as interferometric detection resolve the layer of dye molecules surrounding the microtubules as hollow tubes (see intensity profiles in the Supporting Information). The cavity inside these tubes corresponds well to the outer diameter of microtubules, which is 25 nm^30^.

In the interferometric reconstructions, weaker ghost images of the structures appear in some regions at a distance of about half the wavelength. These artefacts are caused by false assignments of *z* positions due to the similarity of the interferometric PSFs at distances of half the wavelength. Their number was low enough not to impair recognisability of the tubulin strands.

The dye Alexa568 is less bright than Alexa647 and therefore features inferior localisation precision. Accordingly, the ring-shaped structures are less clearly discernible for Alexa568 than for Alexa647 when astigmatic detection was used, and could not be resolved without ambiguity. In contrast, for interferometric detection the localisation precision was superior so that even for Alexa568 the rings were clearly visible.

### Resolving power several *μ*m deep in the sample

**Figure 4:**
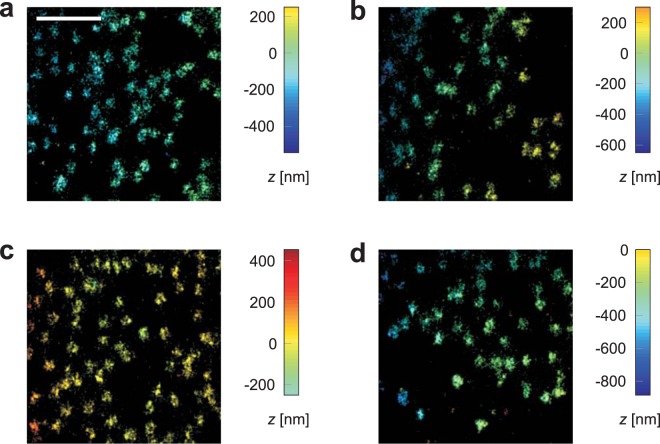
3D dSTORM images of nuclear pore complexes in the nuclear membrane, several *μ*m deep in HeLa cells. Nucleoporin 358 was stained with Alexa647 and measured with astigmatic detection. (**a**) Bottom side of the nucleus, near the cover glass. (**b**) Top side of the same nucleus, at a distance of 6.4 *μ*m to the bottom. (**c**) Second example for the bottom side of a nucleus, near the cover glass. d: Top side of the same nucleus, at a distance of 5.4 *μ*m to the bottom. Scale bar 1 *μ*m.

To show that our setup with refractive index matching permits imaging several *μ*m deep in the sample, we also performed 3D dSTORM with astigmatic detection of nuclear pore complexes (NPCs) in HeLa cells. Here, the nucleoporin 358 (Nup358) was labelled with Alexa647. Nup358 is located at the tip of the highly flexible cytoplasmic filaments of the NPC. In the 3D dSTORM images, the NPCs stained in this way are visible as little pores with diameter ~70 nm (see Fig. 4), which is in the same range as results from electron microscopy measurements for different species^31,32^. The pores can be resolved at the nuclear membrane adherent to the cover glass as well as on the opposite membrane at a distance ≳5 *μ*m to the cover glass, which shows that the resolution is not markedly degraded deep in the sample.

A measurement with glycerol-free buffer of refractive index 1.35 proved that index matching is advantageous for 3D localisation with two objectives (see supporting information). Due to the refractive index mismatch between cover glass and sample buffer, the objective at the opposite side of the sample exhibited a pronounced spherical aberration and yielded inferior results than the objective directly facing the cells, especially for axial localisation.

### Distance measurements with Nanorulers

**Figure 5:**
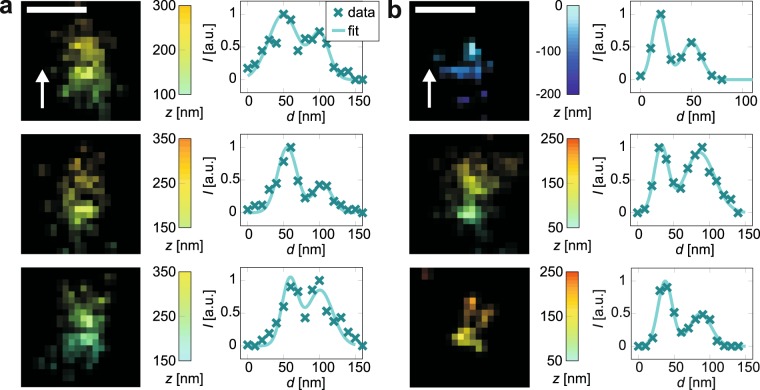
Distance measurements with 3D DNA structures. dSTORM images with corresponding line fits, for (**a**) astigmatic detection, (**b**) interferometric detection. Shown are three examples for each method. Scale bar 100 nm, white arrows indicate the optical axis, *d* is the position along the intensity profile and *I* the normalized intensity.

The axial resolving power in dSTORM measurements was further examined using custom-made GATTAquant Nanorulers, which are convenient tools to test the spatial resolution of fluorescence microscopes. The Nanorulers are artificial DNA structures where two surfaces at an axial distance of (33 ± 3) nm are labelled with Alexa647. One side of the structure is functionalised with biotin so that it couples to cover glasses coated with BSA-biotin and neutravidin such that the Nanoruler is oriented parallel to the optical axis. To investigate if the detection modalities implemented in our microscope could resolve the spatial separation of both surfaces, their distance was measured in intensity profiles created from dSTORM reconstructions (Fig. 5).

After image acquisition and reconstruction, axial slices of the structures were analysed with ImageJ^33^. In these images, the DNA constructs appear elongated in direction of the optical axis. Intensity profiles along their extent were created by summing up all image pixel counts on a width of 100 nm using ImageJ’s Plot Profile function.

In the second step, the intensity profiles were normalized and MATLAB® (The MathWorks, Inc., Natick, Massachusetts, USA) was used to fit the sum of two 1D Gaussian functions

3 $$g(d)=\exp [-\frac{{(d-{p}_{1})}^{2}}{2{p}_{4}^{2}}]+{p}_{3}^{2}\exp [-\frac{{(d-{p}_{2})}^{2}}{2{p}_{5}^{2}}]$$

to these intensity distributions by least squares fitting. *d* is the distance along the 1D intensity plot and *p*_1_ to *p*_5_ are fit parameters. Accordingly, the distance between the two maxima of the plot is *d*_*z*_ = |*p*_2_ − *p*_1_|. Fits *g*(*d*) which deviated so far from the data *I* that ${\chi }^{2}={\sum }_{i}^{{N}_{l}}\,{[{I}_{i}-g({d}_{i})]}^{2}\,/g({d}_{i})$^25^ surpassed the *χ*^2^ value corresponding to a 5% level of significance were excluded from the analysis. *N*_*l*_ is the number of data points of the intensity profile. This was done to ensure that only data sets which could be correctly modelled by two distinct peaks were evaluated.

50 data sets acquired with astigmatic detection were analysed. Fits resulted in an axial distance of *d*_*z*_ = (38 ± 2) nm. The error is the standard deviation of all *d*_*z*_ values divided by the square root of the number of measurements. The result for *d* is slightly greater than the specified value (33 ± 3) nm. This indicates that the distance was close to the resolution of the astigmatic scheme, but could be resolved by this detection method.

When images of 36 structures obtained with interferometric detection were analysed, an axial distance of *d*_*z*_ = (34 ± 2) nm was obtained. Compared to the other *z* detection method, this result is closest to the true value, which confirms the higher resolving power of interferometric detection.

The deviation of the distance measured by astigmatic detection from the true value is probably a systematic error. This error is caused by the inferior resolution of this method, as distances smaller than the resolution cannot be detected and therefore do not contribute to the computation of the mean distance *d*_mean_. If we assume that the measured distances are normally distributed around a mean value *d*_0_ with a standard deviation *s*, this can be seen from an estimate for *d*_mean_:

4 $${d}_{{\rm{m}}{\rm{e}}{\rm{a}}{\rm{n}}}=\{{\int }_{{d}_{min}}^{{d}_{max}}\,\frac{q}{s\sqrt{2\pi }}\,\exp \,[-\frac{{(q-{d}_{0})}^{2}}{2{s}^{2}}]{\rm{d}}q\}/\{{\int }_{{d}_{min}}^{{d}_{max}}\,\frac{1}{s\sqrt{2\pi }}\,\exp \,[-\frac{{(q-{d}_{0})}^{2}}{2{s}^{2}}]{\rm{d}}q\},$$

where the integration limits *d*_min_ and *d*_max_ correspond to the measurement range. Let us assume that *d*_0_ is the unbiased true value *d*_0_ = 33 nm, and that we can measure distances infinitely long, *d*_max_ = ∞, but not smaller than the full width at half maximum calculated from the localisation precision, ${d}_{{\rm{\min }}}=2\sqrt{2\,\mathrm{ln}\,\mathrm{(2)}}{u}_{z}$. The distance is a difference of two positions, so we estimate for the width of its distribution approximately two times the localisation precision, *s* = 2*u*_*z*_. If we interpolate the localisation precisions from the bead measurements to the typical brightness of Alexa647 by multiplying by a factor of $\sqrt{17000/3823}$  (photon number for beads/photon number for Alexa647^26^), we obtain *d*_mean_ = 33 nm for interferometric detection, which means that the result is still unbiased. But for astigmatic detection, the inferior resolution increases the expected distance to *d*_mean_ = 38 nm, which agrees well with the measured result.

## Discussion

We showed that glycerol immersion objectives in conjunction with a glycerol-containing imaging buffer are well suited for dSTORM imaging of biological samples. Systematic comparison of theoretical as well as experimental localisation precisions for astigmatic and interferometric detection revealed that astigmatic 3D detection with two glycerol immersion objectives already yields almost isotropic 3D localisation precisions and resolves structures of about 30 nm in dSTORM experiments without necessitating complicated alignment or specialized optics, making it a convenient choice for biological applications. Adding a second objective improved the localisation precision by $\sqrt{2}$ as predicted. When interferometric detection is employed, the localisation precisions are even better for all three dimensions, but the experimental complexity is increased.

As our setup allows to switch between these detection options, it enables choosing the method for estimation of the axial coordinate according to the required resolution. For many applications, astigmatic detection with two objectives will be sufficient, but when even smaller structures need to be resolved, it is possible to change to interferometric detection.

The microscope presented here can be easily converted into a biplane detection setup, which further enhances the flexibility of the setup. This will allow to analyse the performance of refractive index matching in conjunction with an additional 3D imaging technique (preliminary results not shown).

As we showed by simulations, exact adjustment of the refractive indices improves the localisation precision. Furthermore, it will be especially beneficial for quantitative measurements, for example distance measurements, because it not only reduces distortions of the image field but chromatic errors as well. Furthermore, different refractive indices in sample buffer and immersion medium may lead to erroneous scaling of *z* localisation results due to different contractions of the optical axes. With identical refractive indices, this problem does not occur.

Because aberrations due to refractive index mismatch are more prominent deep in the sample, index matching enables single molecule localisation microscopy of thicker samples. In our measurements, we could image intracellular structures near the cover glass as well as more than 5 *μ*m deep in the sample. The benefit of index matching for 3D single molecule localisation microscopy was also recently demonstrated for water immersion objectives in conjunction with a double-helix PSF^34^. In contrast to water immersion, glycerol immersion enables employing objectives with a higher NA. As the detection schemes presented by us only perform well for a depth of field of about 0.5 *μ*m, thicker samples of several *μ*m require capturing many planes at varying *z* positions. But this is easily feasible by moving the piezoelectric stage with the sample holder, however at the cost of an increased acquisition time.

## Methods

### Optical setup

In our custom-built microscope, photoactivation and fluorescence excitation in three spectral ranges is enabled by four lasers (LuxX 647-140, 647 nm, 140 mW, Omicron Laserage Laserprodukte GmbH, Rodgau, Germany; Cobolt Jive, 561 nm, 100 mW, Cobolt AB, Solna, Sweden; Cobolt Calypso, 491 nm, 100 mW, Cobolt AB, Solna, Sweden; PhoxX 405-60, 405 nm, 60 mW, Omicron Laserage Laserprodukte GmbH, Rodgau, Germany). The light intensity incident on the sample is controlled by an acousto-optical tunable filter (AOTFnC-400.650 with driver MDS4C-B66-22-74.158.RS, AA opto-electronic, Orsay, France). The illumination light beam is widened by a telescope to a diameter of about 6 mm and focused on the back aperture of one of the objectives by an achromat with focal length 200 mm (G063148000, Qioptiq Photonics GmbH & Co. KG, Göttingen, Germany) so that it leaves the objective in a collimated way. Two removable wedge prisms (PS810-A mounted in removable optic holder LM1-A with LM1-B/M, Thorlabs Inc, Newton, New Jersey, USA) allow optional illumination at a highly inclined angle which reduces background fluorescence. The illumination light enters the detection part of the setup via a quadband beamsplitting filter (zt405/488/561/633trans, AHF analysentechnik AG, Tübingen, Germany) which transmits the laser lines but reflects the fluorescence light.

**Figure 6:**
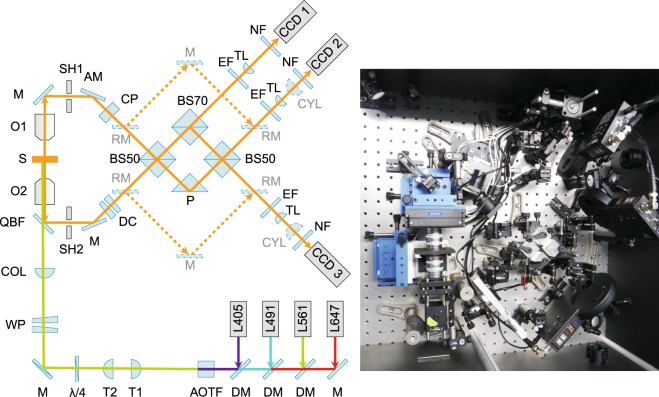
Sketch of the beam path and photo of the detection part. Dashed lines indicate the detection beam path with inserted removable mirrors, which is used for astigmatic detection. AM: adjustable mirror, AOTF: acousto-optical tunable filter, BS50: beamsplitter 50:50, BS70: beamsplitter 70:30 (T:R), CCD1: charge-coupled device 1, CCD2: charge-coupled device 2, CCD3: charge-coupled device 3, COL: collimation lens, CP: compensator plate, CYL: cylindrical lens, DC: dispersion compensator, DM: dichroic mirror, EF: emission filter, L405: laser 405 nm, L491: laser 491 nm, L561: laser 561 nm, L647: laser 647 nm, λ/4:  quarter wave plate, M: mirror, NF: notch filter, O1: objective 1, O2: objective 2, QBF: quadband beamsplitting filter, P: prism, RM: removable mirror, S: sample, SH1: shutter 1, SH2: shutter 2, T1: telescope lens 1, T2: telescope lens 2, TL: tube lens, WP: wedge prisms.

Two glycerol-immersion objective lenses (HCX PL APO 100x/1.35 GLYC CORR, Leica Microsystems, Wetzlar, Germany) capture the fluorescence light emitted by the sample. A glycerol-water mixture with 87% glycerol (G5515-100ML, SIGMA ALDRICH CHEMIE GmbH, Steinheim, Germany) and 13% water (v/v) is a suitable medium for immersion. Objective O1 in Fig. 6 is adjustable along three axes. Coarse positioning is done via a custom-made three-axis linear adjustment unit (R. Rittmeyer GmbH, Münster, Germany) with counter surfaces of sapphire to minimise friction and undesired shift during motion. This unit carries a three-axis piezo nanopositioner (Nano-F3D with Nano-Drive controller and 20 bit USB interface, Mad City Labs Inc., Madison, Wisconsin, USA) for fine adjustment. The sample is mounted onto a three-axis linear adjustment unit with counter surfaces of sapphire (R. Rittmeyer GmbH, Münster, Germany) as well. Fine adjustment along the optical axis with position feedback is required for focusing and for capturing calibration curves for *z* position estimation. This is accomplished by a single-axis piezo nanopositioner (Nano-OP100-M with Nano-Drive controller and 20 bit USB interface, Mad City Labs Inc., Madison, Wisconsin, USA).

Mirrors in removable magnetic mounts (KS1R, Thorlabs Inc, Newton, New Jersey, USA) allow switching between two detection pathways: If the mirrors are inserted, the fluorescence light detected by each objective is directly led to one EMCCD camera (iXon3 897, Andor Technology Ltd., Belfast, UK) where it is focused onto the detector chip by a tube lens with focal length 200 mm (G063205000, Qioptiq Photonics GmbH & Co. KG, Göttingen, Germany). This beam path is employed for astigmatic detection. For this 3D detection method, additional cylindrical lenses with focal length 300 mm (LJ1558RM-A, Thorlabs Inc, Newton, New Jersey, USA) have to be inserted in front of the cameras. They cause an elliptic distortion of the PSF which enables to back-calculate an emitters’ axial position from the width of the PSF.

If the removable mirrors are absent, the light of both objectives is combined by two 50:50-beamsplitters (BS013, Thorlabs Inc, Newton, New Jersey, USA), one 70:30-beamsplitter (BS019, Thorlabs Inc, Newton, New Jersey, USA) and a prism (RAP100C, Laser Components GmbH, Olching, Germany) which may be translated precisely via a piezoelectric linear actuator (8353 with controller 8742-4-KIT, Newport Spectra-Physics GmbH, Darmstadt, Germany). This configuration leads to sinusoidal, *z*-dependent variations of the intensities measured at each camera^8^ and enables to detect the axial coordinate with interferometric precision. As this requires an exact alignment of the detected beams and precise adjustment of the optical path lengths, both the tilt of the beam exiting objective O1 and the path length in the corresponding arm may be controlled by an adjustable mirror. This mirror can be tilted along two axes via a piezoelectric tilt actuator (Nano-MTA2 with Nano-Drive controller and 20 bit USB interface, Mad City Labs Inc., Madison, Wisconsin, USA) and displaced backward and forward by a single axis nanopositioner (Nano-OP30-M with Nano-Drive controller and 20 bit USB interface, Mad City Labs Inc., Madison, Wisconsin, USA). A two-axis linear translation stage with rotation platform (XYR1/M, Thorlabs Inc, Newton, New Jersey, USA) enables coarse positioning of the mirror. A custom-made dispersion compensator consisting of a pair of glass wedges and a compensator plate (Bernhard Halle Nachfl. GmbH, Berlin, Germany) allows to compensate small differences in glass thickness of both arms of the interferometer which arise from manufacturing tolerances of the optical building parts.

In front of the cameras, the laser light is removed by notch filters (QuadLine Rejectionband ZET405/488/561/640, AHF analysentechnik AG, Tübingen, Germany). A set of three emission filters (700/75 ET Bandpass, 617/73 BrightLine HC, 525/50 ET Bandpass, AHF analysentechnik AG, Tübingen, Germany) permits to choose the spectral range of the detected fluorescence.

### Particle localisation algorithm

For obtaining the *x* and *y* coordinates (lateral plane), a 2D Gaussian function was fit to regions of interest containing a single fluorescent molecule. Fitting was effected with maximum likelihood estimation.

Coordinates along the optical axis (*z*) were estimated by comparing properties of the single molecule images to calibration curves. To obtain calibration curves, a sub-resolution fluorescent bead is translated along the optical axis and imaged every 20 nm.

Cylindrical lenses cause an astigmatic distortion. For this detection scheme, a quadratic function with some higher order terms is fit to the width of the bead images.

For interferometric detection, sine curves are fit to the normalized intensities of each detector. To avoid ambiguities, a slight astigmatic aberration present in the system is used for coarse positioning^35^.

The Supplementary Information includes a more detailed description of the particle localisation process.

Spatial misalignments between the image stacks captured by each camera were corrected by determining affine transformations between the images from measurements with brightly fluorescent beads prior to imaging. Bead *x* and *y* positions were obtained as described above and from these sets of corresponding positions, the affine transformations were calculated using functions from MATLAB®’s Image Processing Toolbox (The MathWorks, Inc., Natick, Massachusetts, USA).

Spatial drift during measurements was corrected, if necessary, by localising sub-resolution sized fluorescent beads added to the samples and subtracting their deviation from their position at the beginning of the measurement from the *x*, *y* and *z* coordinates.

### Preparation of bead samples

To prepare a bead sample for estimating the localisation precision, one 30 mm diameter fused silica cover glass (Leica Microsystems, Wetzlar, Germany) was glued into a metal ring (Feinmechanische Werkstatt im Physikalischen Institut, Münster, Germany). First of all, the cover glass in the ring and a second one required as counterpart were treated in a plasma cleaner (Femto with generator 40 kHz, 2000 V, 200 W, Diener electronic GmbH + Co. KG, Elbhausen, Germany). The generator was set to work at medium power (100 W) and processing time (6 min 40 s). After treatment in the plasma cleaner, 50 *μ*l of TransFluoSpheres® (T8861, Invitrogen Ltd., Paisley, UK) emitting at 605 nm diluted 1:10000 in deionized water were pipetted onto the cover glass and incubated for 2 min. Then the cover glass was washed by carefully applying and removing 150 *μ*l of deionized water and let dry. Finally, 15 *μ*l of immersion medium were added onto the cover glass surface, the second cover glass was put on top and the sample was sealed with a two-component-glue based on polydimethylsiloxane (Wirosil® Doublier-Silikon, BEGO Bremer Goldschlägerei Wilh. Herbst GmbH & Co. KG, Bremen, Germany).

### Staining process for microtubules

For imaging microtubules, 3T3 mouse embryonic fibroblast cells were grown on fused silica cover glasses (diameter 18 mm, custom-made by XanTec bioanalytics GmbH, Düsseldorf, Germany). The cells were washed with 500 *μ*l of phosphate-buffered saline (PBS, L182-01, BIOCHROM AG, Berlin, Germany), fixed for 20 min with 4% of paraformaldehyde (P6148, SIGMA ALDRICH CHEMIE GmbH, Steinheim, Germany) in PBS, washed again and then blocked for 20 min with 500 *μ*l of 100 mM glycine (HNO7.3, Carl Roth GmbH + Co. KG, Karlsruhe, Germany) in PBS. After this, they were washed three times and incubated for one hour with PBS containing 3% (w/v) bovine serum albumin (BSA, A9085, SIGMA ALDRICH CHEMIE GmbH, Steinheim, Germany) and 0.2% (w/v) saponin (S7900, SIGMA ALDRICH CHEMIE GmbH, Steinheim, Germany), denoted blocking buffer in the following. Next, the primary antibodies (mouse monoclonal *α*-tubulin, 302 211, Synaptic Systems GmbH, Göttingen, Germany) were applied. They were diluted 1:500 in 250 *μ*l blocking buffer and incubated for one hour.

After removing the antibody solution, the cover glasses were washed five times with PBS with 0.2% (w/v) BSA and 0.05% (w/v) saponin, called washing buffer. Subsequently, the secondary antibodies were applied. For the red spectral range, they were labelled with Alexa647 (goat anti-mouse, A21236, Invitrogen Ltd., Paisley, UK) and for the orange spectral range with Alexa568 (goat anti-mouse, A21207, Invitrogen Ltd., Paisley, UK).

The antibodies were diluted 1:500 in 250 *μ*l of blocking buffer and incubated for one hour. The cover glasses were washed five times with 500 *μ*l washing buffer and one time with PBS, fixed for 10 min with 500 *μ*l of 4% paraformaldehyde and 0.1% glutaraldehyde (glutaraldehyde 25% solution in water, 23114.02, SERVA Electrophoresis GmbH, Heidelberg, Germany) in PBS and washed once again. Finally, the samples were blocked for 20 min with 500 *μ*l of 20 mM glycine in PBS and washed five times with PBS.

### NPC staining

The primary antibody was polyclonal rabbit anti-Nup358 (a gift from E. Coutavas, Rockefeller University of New York) and the secondary antibody Alexa647-labelled goat anti-rabbit (A32733, Invitrogen Ltd., Paisley, UK), otherwise the staining process was identical to the tubulin staining described above.

### Nanoruler sample preparation

To prepare a sample with Nanorulers, a fused silica cover glass (diameter 30 mm, custom-made by XanTec bioanalytics GmbH, Düsseldorf, Germany) was cleaned in the plasma cleaner as described above. After waiting for 20 min (to prevent the surface charges created by the plasma from interfering with the coating process), it was washed with PBS (L182-01, BIOCHROM AG, Berlin, Germany). Then it was incubated for 5 min with 50 *μ*l of a solution of 1 $\frac{{\rm{mg}}}{{\rm{ml}}}$ biotin and bovine serum albumin in PBS (GATTAquant GmbH, Braunschweig, Germany). Next, this solution was removed and the cover glass was again washed with PBS. Then it was incubated for 5 min with 50 *μ*l of 1 $\frac{{\rm{mg}}}{{\rm{ml}}}$ neutravidin in PBS (GATTAquant GmbH, Braunschweig, Germany). After this, the glass surface was washed three times with PBS with 50 mM MgCl_2_ (HNO3.1, Carl Roth GmbH + Co. KG, Karlsruhe, Germany). 0.5 *μ*l of solution containing the DNA construct (GATTAquant GmbH, Braunschweig, Germany) were diluted in 50 *μ*l PBS with 50 mM MgCl_2_ and incubated for 5 min on the cover glass. Again, it was washed two times with PBS with 50 mM MgCl_2_. Then, red fluorescent beads for calibration and drift correction (FluoSpheres® Carboxylate-Modified Microspheres 660/680, F8789, Invitrogen Ltd., Paisley, UK) were diluted 1:3000000 in this buffer, incubated for 2 min and were removed. Finally, the sample was washed once more with PBS with 50 mM MgCl_2_ and mounted with dSTORM buffer supplemented with 50 mM MgCl_2_ (see below).

### dSTORM microscopy of microtubules, NPCs and Nanorulers

For calibration and drift correction, fluorescent beads were added to the samples, red-fluorescing beads for the samples with A647 (FluoSpheres® Carboxylate-Modified Microspheres 660/680, F8789, Invitrogen Ltd., Paisley, UK) and orange-fluorescing beads for the samples with A568 (FluoSpheres® Carboxylate-Modified Microspheres 580/605, F8793, Invitrogen Ltd., Paisley, UK). The beads were diluted 1:1000000 in 500 *μ*l PBS and incubated on the sample for 2 min. Then the sample was washed another three times with 500 *μ*l PBS. In measurements with NPCs, we used TetraSpeck® Microspheres (T7279, Invitrogen Ltd., Paisley, UK). They were diluted 1:100 in 250 *μ*l PBS and incubated on the sample for 5 min. Prior to imaging, all samples was embedded in dSTORM buffer (see below).

For dSTORM, samples were embedded in imaging buffer based on a buffer containing 200 mM 1,4-piperazinediethanesulfonic acid (A1079, AppliChem GmbH, Darmstadt, Germany), supplied with 40% glucose (6887.1, Carl Roth GmbH + Co. KG, Karlsruhe, Germany) and adjusted to a pH of 7.2. Glycerol (G5515-100ML, SIGMA ALDRICH CHEMIE GmbH, Steinheim, Germany) was added to the buffer until its refractive index was in the range of 1.45. The glycerol concentration then was about 55% of volume. The refractive index was measured with a refractometer (DR201-95, A. Krüss Optronic, Hamburg, Germany). Directly before applying the buffer to the sample, it was supplied with 0.8 $\frac{{\rm{mg}}}{{\rm{ml}}}$ glucose oxidase (G2133-10KU, SIGMA ALDRICH CHEMIE GmbH, Steinheim, Germany), 0.08 $\frac{{\rm{mg}}}{{\rm{ml}}}$ catalase (C1345-1G, SIGMA ALDRICH CHEMIE GmbH, Steinheim, Germany) and 10 mM MEA (also known as cysteamine, 30070-10 G, SIGMA ALDRICH CHEMIE GmbH, Steinheim, Germany).

For imaging, 15 *μ*l of dSTORM imaging buffer were added to the samples and they were mounted with a second cover glass onto a custom metal sample holder in the same way as the bead samples.

For each sample, the correction rings of the objective lenses were adjusted to its refractive index by rotating the rings until the brightness of fluorescent beads in the sample was maximal. Then, calibration curves were obtained. In measurements with Alexa647, the AOTF transmission of the 647 nm laser illumination was set to 100%. This corresponds to an intensity of about $8.6\,\frac{{\rm{kW}}}{{{\rm{cm}}}^{2}}$ in the focal plane. When Alexa568 was used, the transmission for 561 nm was 100%, corresponding to about $7.3\,\frac{{\rm{kW}}}{{{\rm{cm}}}^{2}}$ in the focal plane. For this dye, illumination with the violet diode laser, whose power was reduced to 15 mW, was employed to mediate photoswitching. The respective AOTF transmission was increased from 2% to 15% during measurements in an exponential fashion, augmenting the intensity in the focal plane from about $0.9\,\frac{{\rm{W}}}{{{\rm{cm}}}^{2}}$ to $24\,\frac{{\rm{W}}}{{{\rm{cm}}}^{2}}$. For every dSTORM reconstruction, 60000 frames with an acquisition time of 30 ms were acquired.

We found that in experiments with NPCs, the background was higher at the beginning of the image acquisition, so we discarded the first ~16000 frames for data analysis.

## Electronic supplementary material

Supplementary Information

## Data Availability

The datasets generated during and analysed during the current study are available from the corresponding authors on reasonable request.
