# Supplementary material for: Intrinsic refractive index matched 3D dSTORM with two objectives: Comparison of detection techniques
Source: Sci Rep. 2018 Sep 6;8:13343. doi: 10.1038/s41598-018-31595-z (PMC6127109; doi:10.1038/s41598-018-31595-z)
Supplement: Supplementary file 1 — Supplementary Information [file 41598_2018_31595_MOESM1_ESM.pdf]

Intrinsic refractive index matched 3D dSTORM with two objectives: Comparison of detection techniques

Nora C. Schmidt[1,2], Martin Kahms[2], Jana Hüve[1,2,*], and Jürgen Klingauf[1,2,3,*]

[1]Fluorescence Microscopy Facility Münster (FM)[2], Institute of Medical Physics and Biophysics, Center For NanoTechnology (CeNTech), Heisenbergstraße 11, 48149 Münster, Germany
[2]Department of Cellular Biophysics, Institute of Medical Physics and Biophysics, University of Münster, Robert-Koch-Straße 31, 48149 Münster, Germany
[3]IZKF Münster and Cluster of Excellence EXC 1003, Cells in Motion, CiM, 48149 Münster, Germany
[*]jana.hueve@uni-muenster.de, klingauf@uni-muenster.de

Supplementary Information

Estimation of the axial coordinate (z)

Astigmatic detection

For astigmatic detection, 2D Gaussian functions were fit to single emitter images. Data acquired by each of the two objectives was analysed independently. Calibration functions

$$s_{cx,y}(z) = p_{1x,y}\left[1+\frac{(z-p_{2x,y})^2}{p_{3x,y}^2}+p_{4x,y}\frac{(z-p_{2x,y})^3}{p_{3x,y}^3}+p_{5x,y}\frac{(z-p_{2x,y})^4}{p_{3x,y}^4}\right]^{1/2} \tag{1}$$

were fit to the standard deviations s_x and s_y for both data sets (see Fig. 1 for an example). p_{1x} to p_{5x} and p_{1y} to p_{5y} are fit parameters.

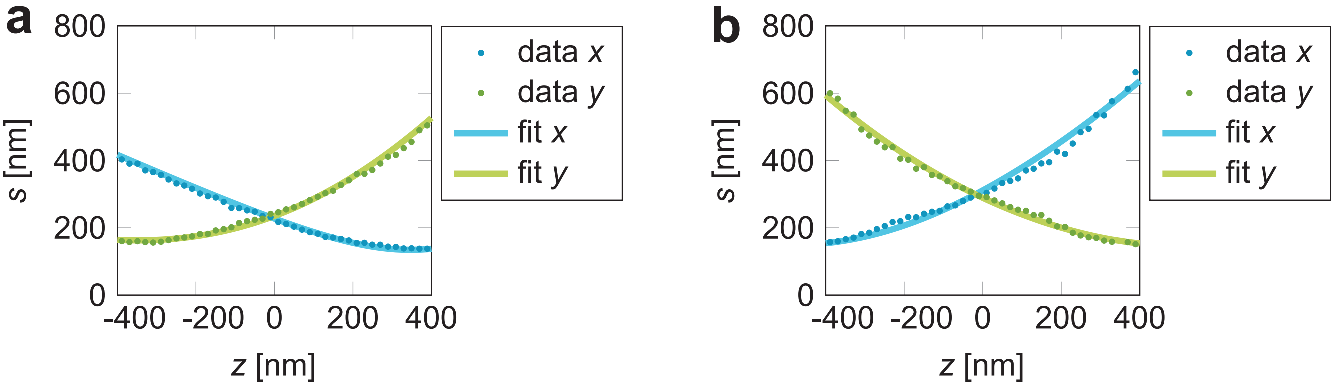

Figure 1. Calibration for astigmatic detection. a: PSF widths for objective 1. b: PSF widths for objective 2.

z positions were estimated for both objectives by fitting these calibration curves to measured x and y standard deviations via obtaining

$$\min_z\left[\left(\sqrt{s_{cx}(z)}-\sqrt{s_x}\right)^2+\left(\sqrt{s_{cy}(z)}-\sqrt{s_y}\right)^2\right]^{1/2} \tag{2}$$

and

$$\min_z\left\{\left[\frac{s_x}{s_y}\right]^{1/2}-\left[\frac{s_{cx}(z)}{s_{cy}(z)}\right]^{1/2}\right\}^2. \tag{3}$$

Then the mean of both z estimations was calculated. Finally, the mean z value for both objectives was determined.

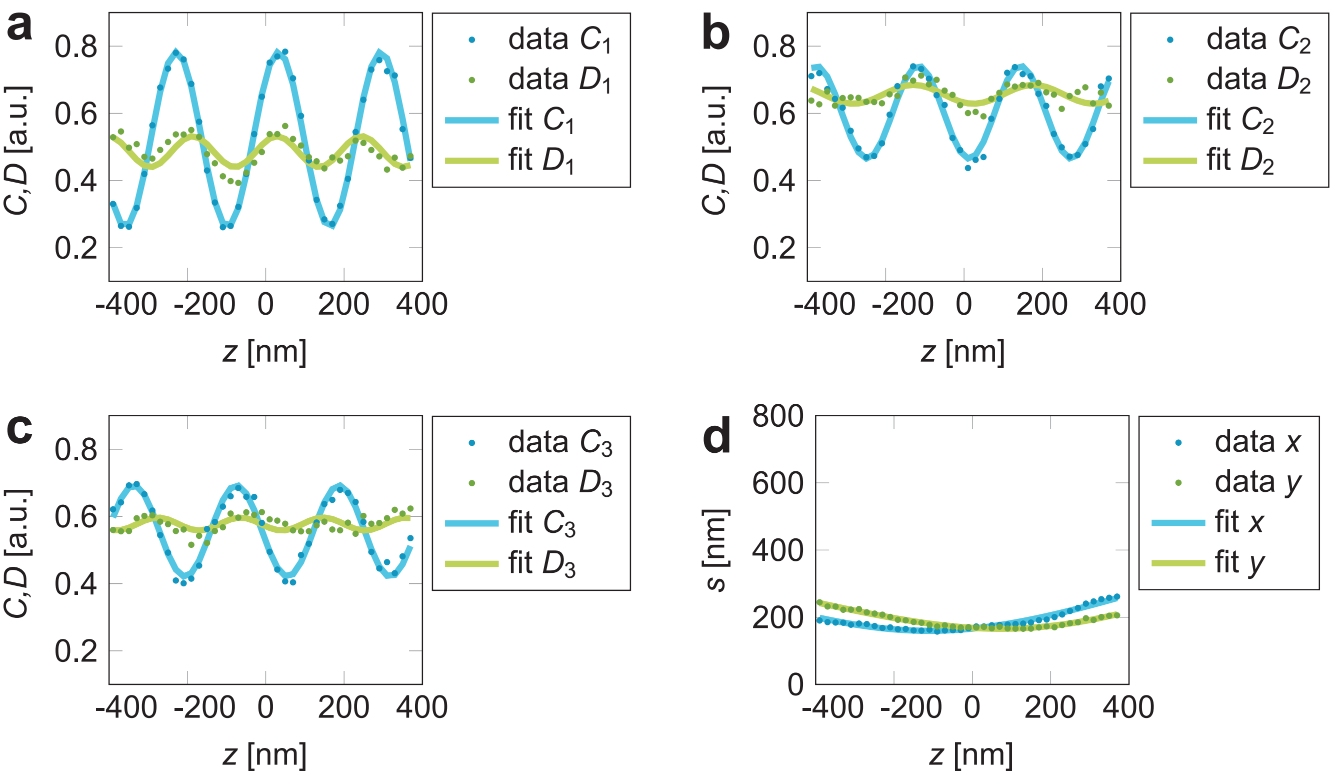

Figure 2. Calibration for interferometric detection. a: Intensity calibration for CCD 1. b: Intensity calibration for CCD 2. c: Intensity calibration for CCD 3. d: Additional astigmatic calibration.

To obtain calibration curves for interferometric detection, a central intensity C

$$C = \sum_{x,y=1}^{N_i} \exp\left[-\frac{(x-c_x)^2 + (y-c_y)^2}{s_0^2}\right] I_{xy}. \tag{4}$$

and a decentral intensity D

$$D = \sum_{x,y=1}^{N_i} \exp\left(-\frac{\left\{\left[(x-c_x)^2 + (y-c_y)^2\right]^{1/2} - r_0\right\}^2}{s_0^2}\right) I_{xy} \tag{5}$$

were calculated for single emitter images acquired with each of the three cameras. N_i is the image's edge length in pixels, c_x and c_y are the molecule's x and y coordinates, $s_0 = 0.6\lambda/[2n_1\sin(\alpha)]$ and $r_0 = 1.7\lambda/[2n_1\sin(\alpha)]$ where λ is the wavelength and n_1 the refractive index of the sample. The values employed here for s_0 and r_0 were found to yield good results. C and D were normalized by

$$C_i = C_i\left(C_1^2 + C_2^2 + C_3^2\right)^{-1/2},\ D_i = D_i\left(D_1^2 + D_2^2 + D_3^2\right)^{-1/2},\ i = 1,2,3. \tag{6}$$

Finally, sinusoidal curves $C_{ci}(z), D_{ci}(z)$ were fit to the normalized intensities (see Fig. 2 for an example).
Interferometric z positions were retrieved from these calibration curves by computing

$$
\begin{aligned}
\min_z \Big\{ & \left[\sqrt{C_1} - \sqrt{C_{c1}(z)}\right]^2 + \left[\sqrt{C_2} - \sqrt{C_{c2}(z)}\right]^2 \\
& + \left[\sqrt{C_3} - \sqrt{C_{c3}(z)}\right]^2 + w\left[\sqrt{D_1} - \sqrt{D_{c1}(z)}\right]^2 \\
& + w\left[\sqrt{D_2} - \sqrt{D_{c2}(z)}\right]^2 + w\left[\sqrt{D_3} - \sqrt{D_{c3}(z)}\right]^2 \Big\}^2
\end{aligned} \tag{7}
$$

where w is a weight constant which was either set to 1 or 2 for all examples shown here. To overcome the problem of ambiguity, coarse z positions z_{asti} were obtained from sum images of all three detectors by analyzing the x and y standard deviations of 2D Gaussian fits as described above. If $|z - z_{\text{asti}}| > 0.5 \cdot z_p$ with $z_p = \frac{2\pi}{p_{21}}$, an offset was added to z. For $z < z_{\text{asti}}$, z was set to $z + z_p$ and for $z > z_{\text{asti}}$ to $z - z_p$.

dSTORM reconstructions

To create images from the lists of coordinates, a 2D Gaussian function was plotted at each localisation's position. In overview images showing larger regions, the contrast of the final images was adjusted linearly to improve visibility.

STED imaging of biological samples

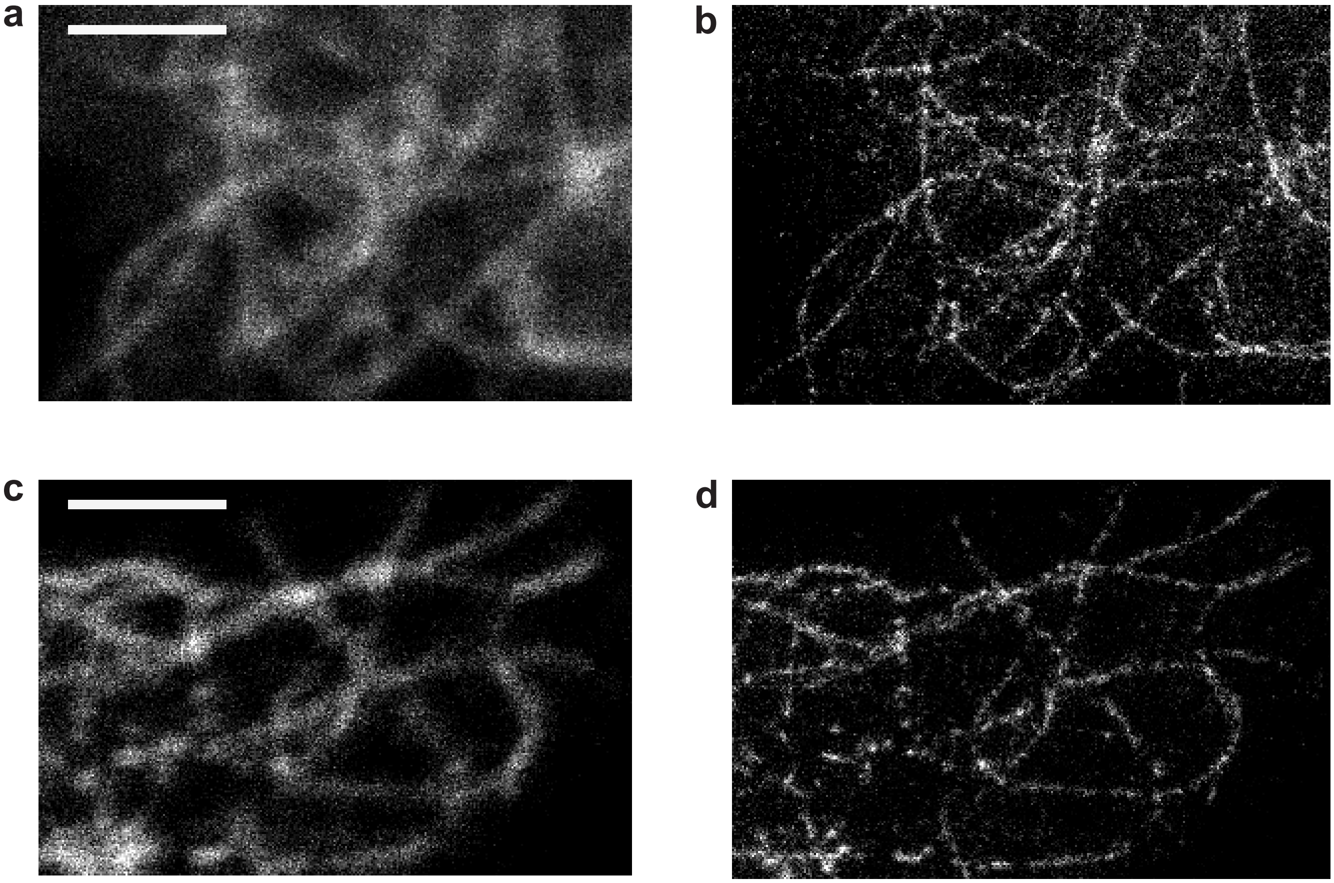

Figure 3. Microtubuli imaged with STED. a: Confocal image for Abberior STAR 635P. b: STED image for Abberior STAR 635P. c: Confocal image for Abberior STAR 580. d: STED image for Abberior STAR 580. Scale bars 2 μm.

For STED imaging (Fig. 3), 3T3 cells were grown on borosilicate cover glasses (No. 1, 1001/18, Glaswarenfabrik Karl Hecht GmbH & Co KG, Sondheim/Rhön, Germany). Microtubules were stained as for dSTORM. Here, secondary antibodies labeled either with Abberior STAR 635P (goat anti-mouse, 2-0002-007-5, Abberior Instruments GmbH, Göttingen, Germany) or Abberior STAR 580 (goat anti-mouse, 2-0002-005-1, Abberior Instruments GmbH, Göttingen, Germany) were used for staining in the red and orange spectral range. Prior to imaging, the samples were embedded in polyvinyl alcohol (MOWIOL®

4-88, Merck KGaA, Darmstadt, Germany). Imaging took place at a Nikon Ti-E microscope (Nikon Instruments Inc., Melville, New York) equipped with a Nikon oil immersion objective of NA 1.45 (Plan Apo λ 100X, Nikon Instruments Inc., Melville, New York) and a STED unit (STEDYCON Supercompact Nanoscope and Confocal Microscope, 13812, Abberior Instruments GmbH, Göttingen, Germany).

Localisation precision for an emitter of realistic brightness

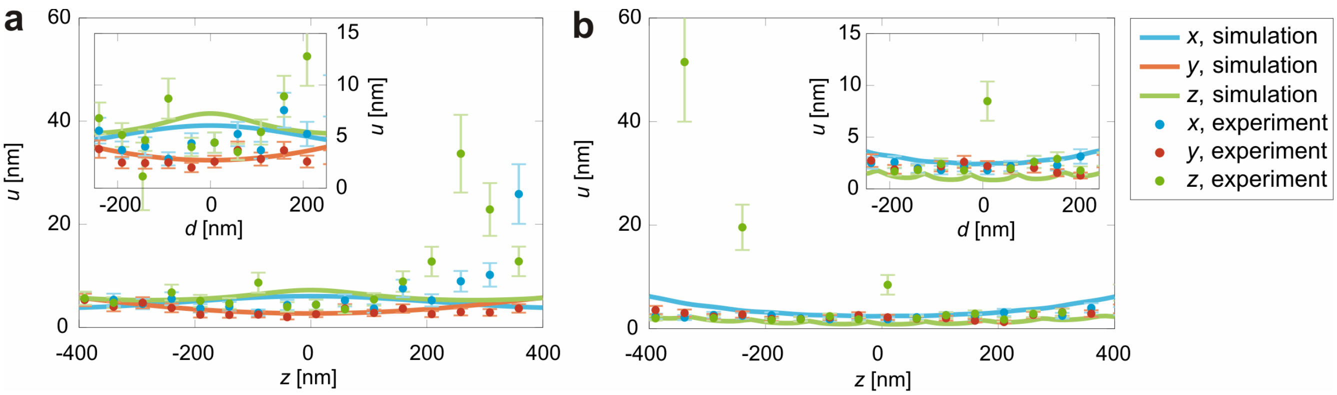

Figure 4. Localisation precisions u against axial position z with emitters of realistic brightness for x, y and z. a: Astigmatic detection. b: Interferometric detection. Insets show magnifications of half a micrometer around the focus. For interferometric detection, simulated localisation precisions are identical for x and y.

To obtain an estimate for the localisation precisions under more realistic conditions, the precision was measured for fluorescent beads fluorescing at a brightness comparable to that of organic (d)STORM dyes. Samples with beads of diameter of 40 nm, emitting at 605 nm (FluoSpheres® Carboxylate-Modified Microspheres 580/605, F8793, Invitrogen Ltd., Paisley, UK) were prepared as described in the Methods section. During acquisition, the incident laser power of the 561 nm laser was adjusted at the AOTF so that about 5000 photons were captured per frame and objective, a value that is comparable to the number of photons emitted per switching cycle by a bright (d)STORM dye[1]. For astigmatic detection, we measured with our dual-objective microscope $u_x = (4.7 \pm 0.3)$ nm, $u_y = (2.9 \pm 0.2)$ nm and $u_z = (6.4 \pm 0.5)$ nm. The results for interferometric detection were $u_x = (2.3 \pm 0.2)$ nm, $u_y = (2.0 \pm 0.1)$ nm and $u_z = (4.5 \pm 0.3)$ nm.

The results were again compared to localisation precisions obtained from a simulated detection PSF. Assumed were $N_s = 5000$ photons per objective detected from the point source and 10 photons per pixel as background (Fig. 4). For the range near focus $z \in (-250\,\text{nm}, 250\,\text{nm})$ the simulation predicts on average $u_x = 5.6$ nm, $u_y = 3.1$ nm and $u_z = 6.1$ nm for astigmatic detection and $u_x = 2.8$ nm, $u_y = 2.8$ nm and $u_z = 1.2$ nm for interferometric detection.

While interferometric detection yields better results, the experimentally measured improvement compared to astigmatism is not as great as predicted theoretically. This may be due to effects of noise.

Localisation precision, half-aperture angle and sample depth

Fig. 5a shows the impact of refractive index mismatch on the localisation precision. The limit of the lateral localisation precision u_{xy} was calculated from simulations of the PSF for an half-aperture angle $\alpha = 60°$, $N = 2826$ collected photons and a hypothetical background of $N/1000$ photons per camera pixel of size $160\,\text{nm} \times 160\,\text{nm}$. Results are shown for an oil immersion objective focusing into a watery sample ($n_s = 1.33$, $n_1 = 1.52$) with emitter positions in the sample $d_s = 1\,\mu$m and $d_s = 10\,\mu$m, for an oil immersion objective without refractive index mismatch ($n_s = n_1 = 1.52$, $d_s = 0$) and for a glycerol objective ($n_s = n_1 = 1.46$, $d_s = 0$). As the refractive indices for glycerol immersion are routinely matched in our experiments and therefore the emitter position d_s has no impact, only the case with $d_s = 0$ is plotted for glycerol. Although due to the higher refractive index of oil, the oil immersion objective has a higher NA compared to the glycerol immersion objective (1.32 versus 1.26), without refractive index mismatch the mean localisation precisions on 0.5 μm around the focus are nearly identical ($u_{xy} = 3.1$ nm for both cases). For the oil immersion objective, a refractive index mismatch degrades the localisation precision depending on the emitter position in the sample ($u_{xy} = 3.5$ nm at a depth of $d_s = 1\,\mu$m in the sample and $u_{xy} = 4.2$ nm at $d_s = 10\,\mu$m depth). Fig. 6 shows the relation between u_{xy} and d_s.

The refractive index mismatch between cover glass and sample for oil immersion objectives could be reduced by adding glycerol (or other suitable substances) to the imaging buffer. In contrast to glycerol immersion, aberrations will still be present as the refractive index of glycerol is too low to achieve complete matching with oil, but they will be decreased.

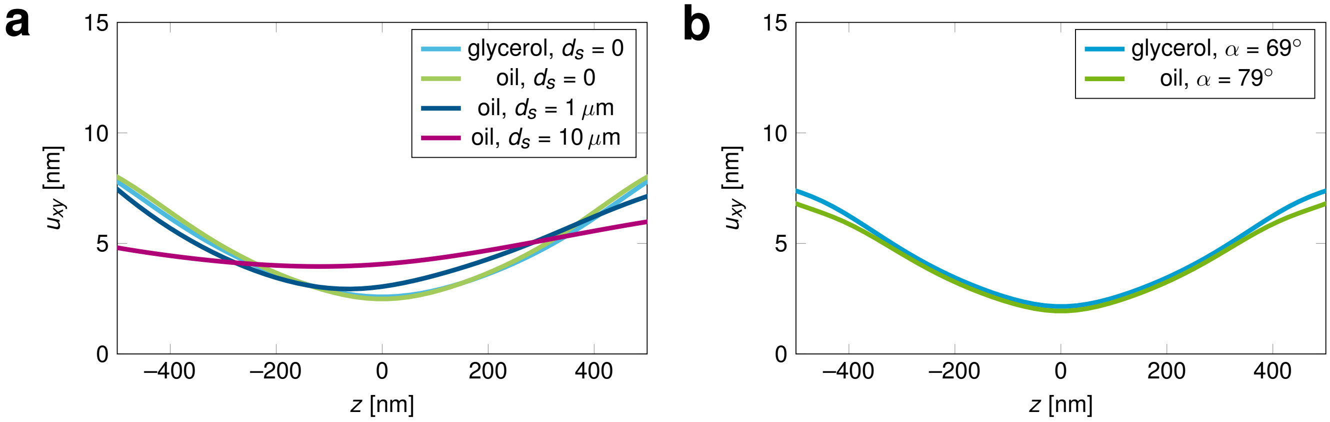

Figure 5. Limit of lateral localisation precision u_{xy} against axial position z. a: With and without refractive index mismatch at two different emitter positions, for an half-aperture angle $\alpha = 60^\circ$ and 2826 collected photons. b: Localisation precisions for an oil-immersion objective with half-aperture angle $\alpha = 79^\circ$ and 4535 collected photons, as well as a glycerol-immersion objective with half-aperture angle $\alpha = 69^\circ$ and 3581 collected photons.

It is true, however, that oil immersion objectives are available with higher half-aperture angles α than glycerol immersion objectives. Consider for example a high-NA oil immersion objective used in our group[2] for 3D dSTORM with a single objective with NA = 1.49 and $\alpha = 79^\circ$, whereas our glycerol immersion objective has NA = 1.36 and $\alpha = 69^\circ$. A higher half-aperture angle leads to a smaller PSF and allows to collect photons from a larger solid angle, which is advantageous for the localisation precision. Therefore it is conceivable that these advantages cancel out degradations by the refractive index mismatch. Unfortunately, our simple approach to describe the mismatch-induced aberration solely as an additional phase breaks down for such large angles of incidence as it neglects the deflection of the electrical fields at the glass-water interface. For a precise calculation of the PSF in this case, a more accurate theoretical model would be required which treats the glass-water interface as an additional optical element.

Nevertheless, it is possible to estimate the impact of a larger half-aperture angle α for the case without refractive index mismatch. We simulated PSFs for the oil ($\alpha = 79^\circ$, $n_1 = 1.52$) and for the glycerol immersion objective ($\alpha = 69^\circ$, $n_1 = 1.46$) and assumed that the number of collected photons N is proportional to the solid angle $\Omega = 2\pi\,[1-\cos(\alpha)]$ spanned by α. If we

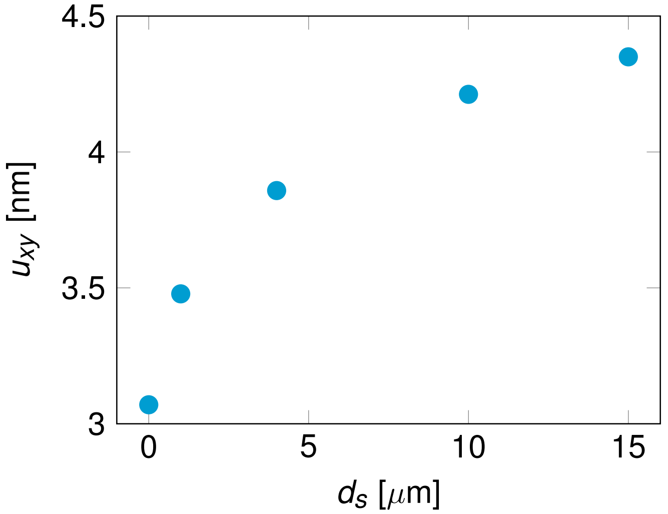

Figure 6. Lateral localisation precision u_{xy} versus emitter position in sample d_s. Localisation precisions were calculated for an oil-immersion objective with half-aperture angle $\alpha = 60^\circ$ focusing into an aqueous sample. u_{xy} is the mean value on $0.5\,\mu$m around the focus. As the focus is shifted due to the refractive index mismatch, it was defined as the axial position where the highest brightness is detected.

have 2826 photons for $\alpha = 60°$, this means that we obtain 3581 photons for $\alpha = 69°$ and 4535 photons for $\alpha = 79°$. Including this into our respective calculations for the limits of localisation precision (Fig. 5b), we have $u_{xy} = 2.8$ nm for the glycerol immersion objective and $u_{xy} = 2.6$ nm for the oil immersion objective, yielding an advantage of 8% for the oil immersion objective. As the effect of the higher NA is quite small, we expect that near the cover glass the oil immersion objective will indeed perform slightly better, but already at distances in the μm range this advantage will be cancelled out by the aberration due to the refractive index mismatch.

For our microscope with two objectives, index matching is especially advantageous as here light is collected via two cover glasses with a distance of about 20 μm. Accordingly, even if the emitter is near one cover glass, it is more than 10 μm away from the other cover glass, meaning that the images acquired by one of the objectives are be perceptibly aberrated.

Intensity profiles of microtubule slices

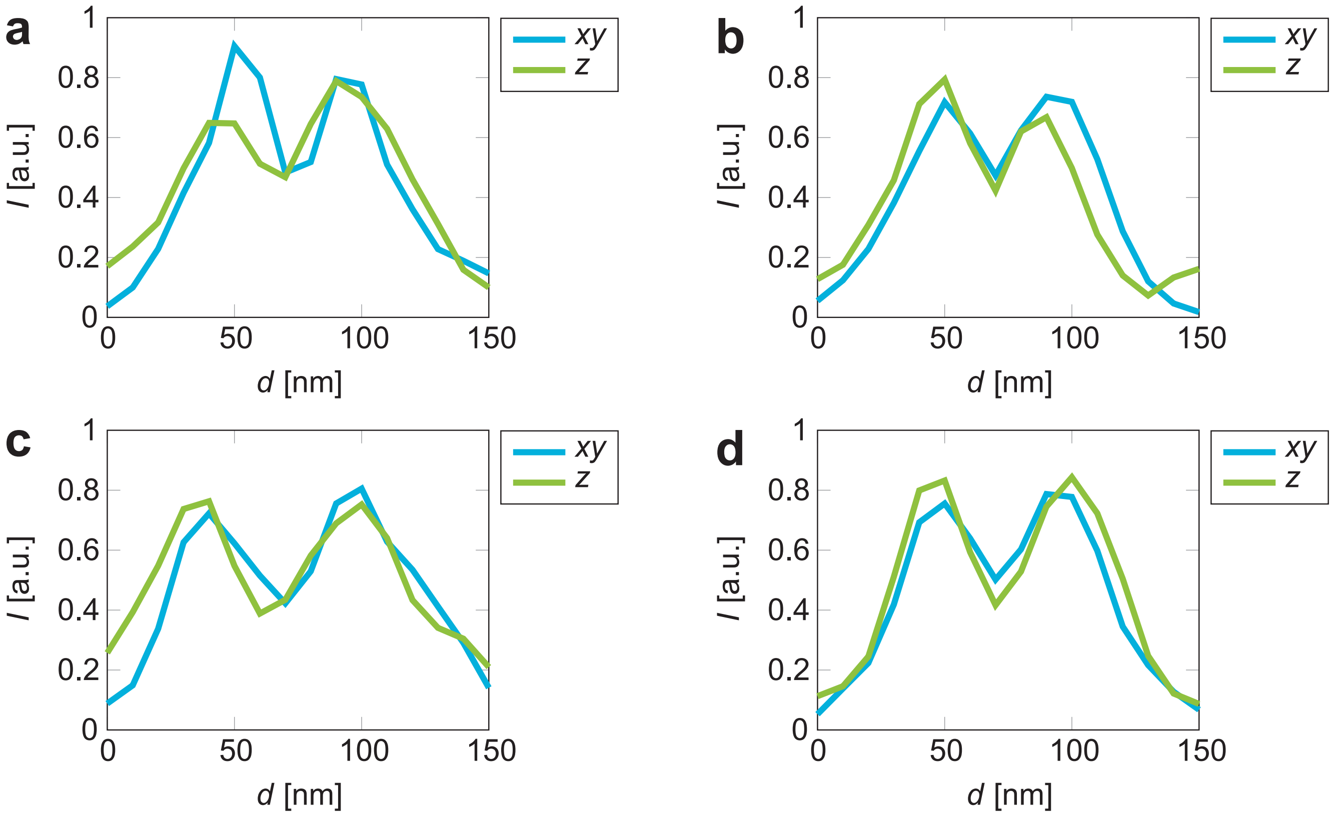

Figure 7. Normalised intensity plots of slices through microtubules. Displayed are averaged curves obtained from averaging the intensity profiles of eight slices per measurement, for the lateral (xy) direction and along the optical axis (z). a: Alexa647, astigmatic detection. b: Alexa647, interferometric detection. c: Alexa568, astigmatic detection. c: Alexa568, interferometric detection.

Fig. 7 shows averaged intensity profiles of transversal slices through microtubules, along the optical axis (z) and perpendicular to the optical axis (xy). The intensity profiles were obtained from reconstructed dSTORM images where each localisation was displayed as a 2D Gaussian function with standard deviation 6 nm for Alexa647 and 7 nm for Alexa568 at its respective position (it was necessary to choose wider spots for Alexa568 because here the labelling appeared sparser). For each slice, all localisations along a mean depth of about 200 nm were summed up. These reconstructions were loaded into ImageJ[3] and plots of the intensity were acquired using ImageJ's Plot Profile function. The profiles were subsequently normalised by dividing by the maximal value. The plots in Fig. 7 display the average of eight such profiles. The hole in the middle, where no antibodies are located, is clearly visible. The distances between the two maxima of the intensity profiles are 40 nm to 60 nm, which agrees well with microtubules decorated with antibodies[4].

Fig. 8 (lateral, xy direction) and Fig. 9 (axial, z direction) show the individual profiles from which the averaged profiles were calculated. Here, the dip in the middle of the intensity profile is also visible in most cases, but the plots look more irregular and often one of the two peaks is much more pronounced than the other. This may be due to sparse labelling, a large displacement of the fluorophores due to the size of the antibodies or a low detection efficiency, which lead to apparent gaps in the rings.

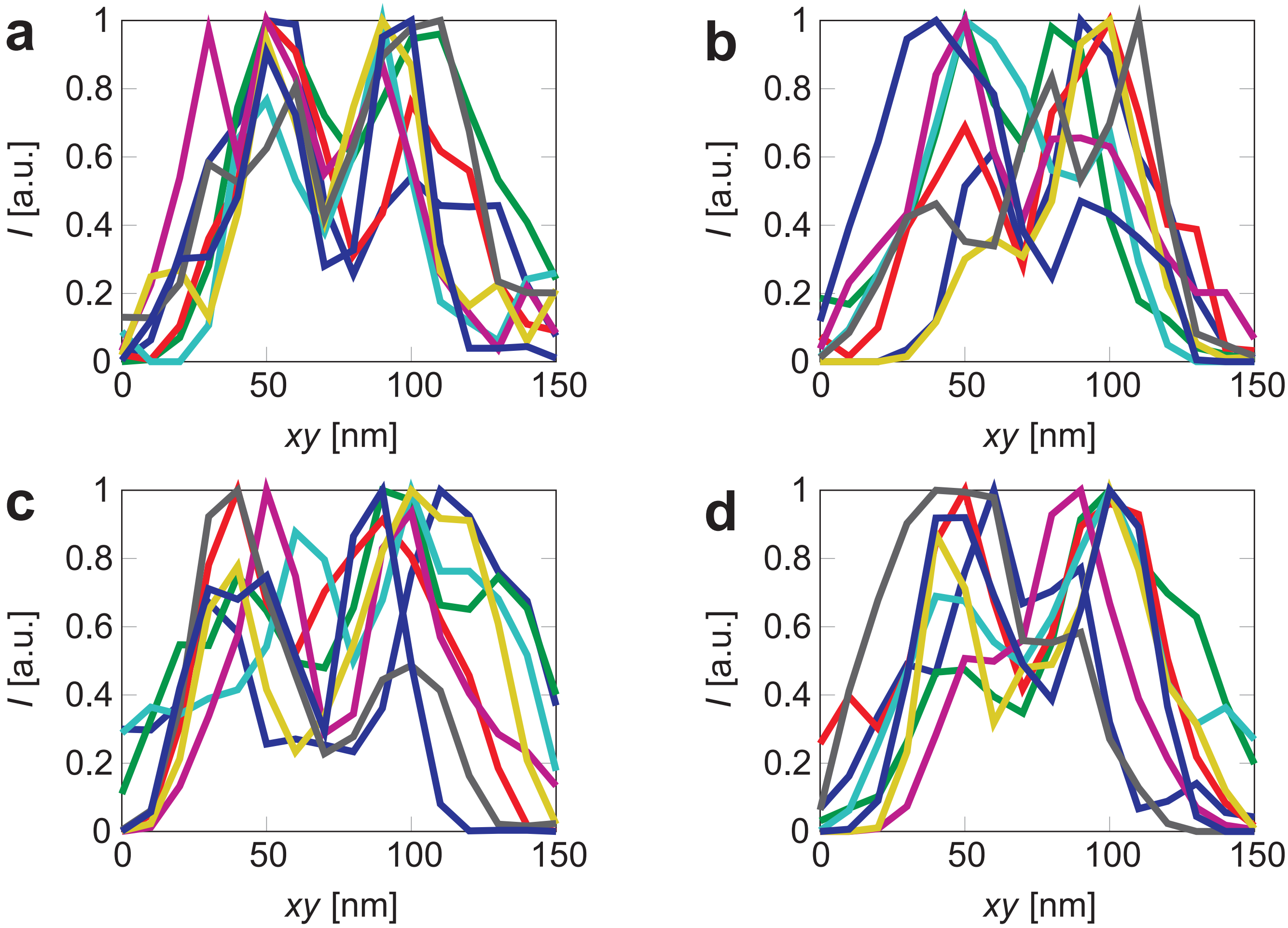

Figure 8. Normalised intensity plots of slices through microtubules. Displayed are intensity profiles of eight slices per measurement, for the lateral (*xy*) direction. a: Alexa647, astigmatic detection. b: Alexa647, interferometric detection. c: Alexa568, astigmatic detection. c: Alexa568, interferometric detection.

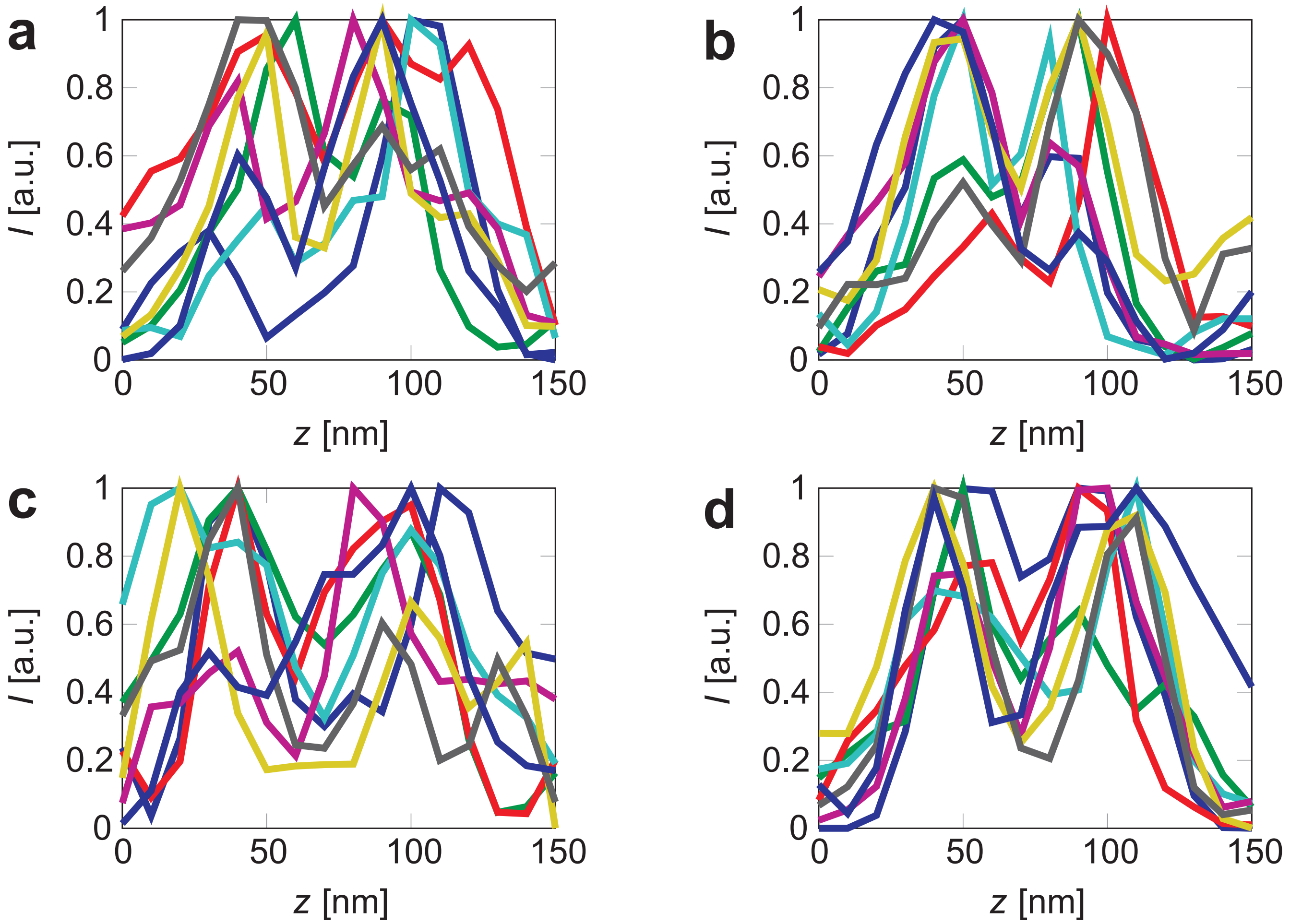

Figure 9. Normalised intensity plots of slices through microtubules. Displayed are intensity profiles of eight slices per measurement, for the direction along the optical axis (z). a: Alexa647, astigmatic detection. b: Alexa647, interferometric detection. c: Alexa568, astigmatic detection. c: Alexa568, interferometric detection.

Switching behaviour of dSTORM dye in glycerol-containing buffer

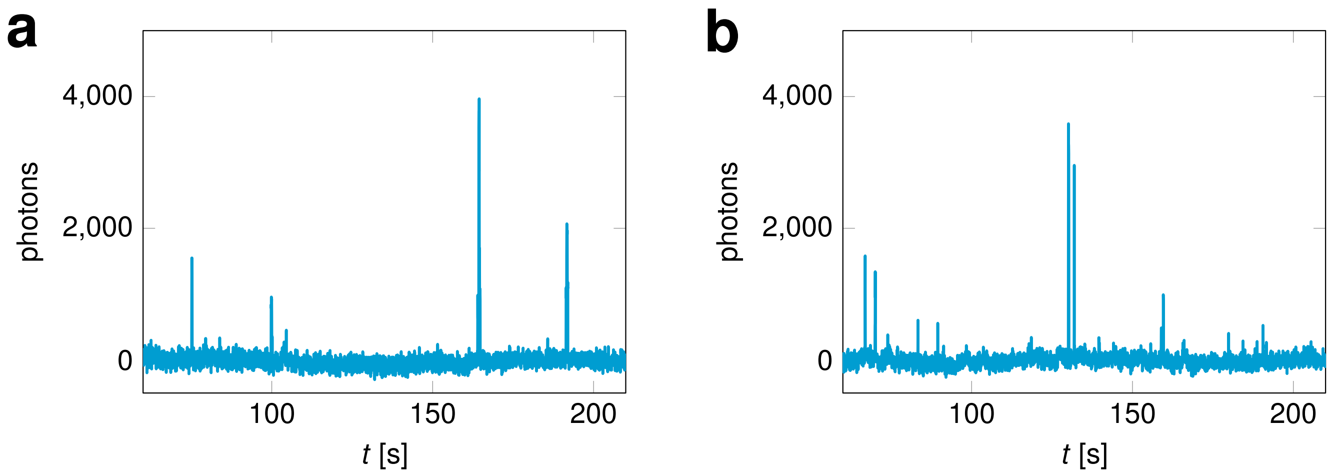

Figure 10. Example for fluorescence time traces of an Alexa647-labelled antibody. a: dSTORM with glycerol-containing dSTORM buffer. b: dSTORM in buffer without glycerol.

To investigate whether a substantial amount of glycerol in the dSTORM buffer affects the switching behaviour, we analysed fluorescence time traces of Alexa647-labelled antibodies. Acquisition conditions were as for dSTORM imaging.

For each time trace, the photons per frame were calculated as the total signal from a region of interest of 800 nm × 800 nm (5 pixels × 5 pixels) minus a time-dependent background B with

$$B(t) = A_1 \exp\left[-\frac{t-t_1}{\tau_1}\right] + A_2 \exp\left[-\frac{t-t_2}{\tau_2}\right] + b \quad (8)$$

(A_1, A_2, t_1, t_2, τ_1, τ_2 and b are fit parameters). The double-exponential function was chosen because it described the data best. For the glycerol-containing buffer, eight traces with a duration of 435 s were analysed and compared to nine traces acquired without glycerol on another custom 3D dSTORM setup[2], employing an oil-immersion objective and standard N-BK7 cover glasses. The glycerol-free buffer was used routinely in our lab for dSTORM and contained 200 mM 1,4-piperazinediethanesulfonic acid adjusted to a pH of 7.0, supplied with 44% glucose, 0.8 $\frac{\text{mg}}{\text{ml}}$ glucose oxidase, 0.08 $\frac{\text{mg}}{\text{ml}}$ catalase and 150 mM MEA.

A molecule was defined as fluorescing when the signal was higher than five times the standard deviation of the trace[1]. For the glycerol-containing buffer, we recorded 185 switching events with a mean on-time of 138 ms and an average of 10600 photons for one objective. Without glycerol, we recorded 203 switching events with a mean on-time of 113 ms and an average of 6000 photons for one objective, indicating that glycerol does not negatively impact the brightness and on-time.

Each of the antibodies used here is typically labelled with two to eight fluorophores[5]. Accordingly, this data set does not allow to determine the number of duty cycles a single dye molecule undergoes before bleaching and the exact duty cycle (fraction of time the molecule is in the fluorescent state). The duty cycle was estimated calculating the fraction of time the antibodies were fluorescing and dividing by five, the expected mean number of fluorophores per antibody. The results are 0.0013 for glycerol-containing buffer and 0.00085 for buffer without glycerol, which is in a similar range as previously reported results[1]. However, for a more accurate investigation of the switching properties, an analysis with single dye molecules adhered to the cover glass is necessary. This will be done in future work.

Impact of refractive index mismatch on 3D localisation

To demonstrate the impact of a refractive index mismatch on the performance of our dual-objective setup, we imaged NPCs in 50 mM Tris buffer at pH 8.0, supplied with 10% glucose (6887.1, Carl Roth GmbH + Co. KG, Karlsruhe, Germany), 0.8 $\frac{\text{mg}}{\text{ml}}$ glucose oxidase (G2133-10KU, SIGMA ALDRICH CHEMIE GmbH, Steinheim, Germany), 0.08 $\frac{\text{mg}}{\text{ml}}$ catalase (C1345-1G, SIGMA ALDRICH CHEMIE GmbH, Steinheim, Germany) and 10 mM MEA (30070-10G, SIGMA ALDRICH CHEMIE GmbH, Steinheim, Germany). This imaging buffer did not contain any glycerol and had a refractive index of 1.35.

The sample was mounted so that cover glass with the cells faced objective O2, whereas objective O1 focused on the cells from the opposite side. Accordingly, light emitted by the fluorophores had to traverse the sample buffer first (typical thickness 20 μm) before being detected by O1, which lead to a pronounced spherical aberration at O1. This aberration dominated the

(purposefully introduced) astigmatic aberration caused by the cylindrical lens, resulting in flatter calibration curves (compare Fig. 11 a and b) and worse axial localisation. This is visible in axial reconstructions created from localisation results calculated independently from data obtained by O1 (Fig. 11 c) and O2 (Fig. 11 d). The degradation is not visible in images obtained by O2 because here the distance between fluorophores and cover glass was only a few hundred nm.

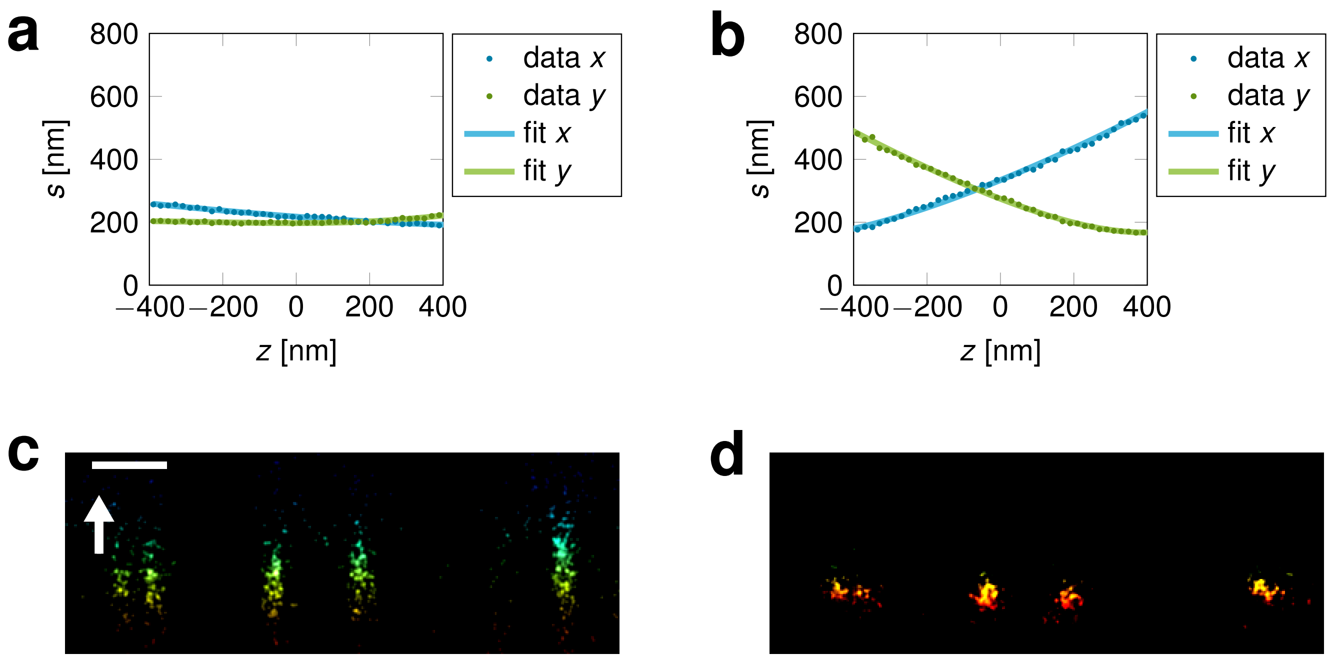

Figure 11. NPCs stained with Alexa647, measured with astigmatic detection, in buffer without glycerol. The HeLa cells where adhered to the cover glass at objective O2, whereas objective O1 imaged the cells from the other side of the sample, resulting in a pronounced spherical aberration. a: Calibration curves for objective O1. Refractive index mismatch supresses the induced astigmatism and leads to flat calibration curves. b: Calibration curves for objective O2. c: Axial slices of NPCs, viewed through Objective O1. Refractive index mismatch degrades the z localisation precision. d: Axial slices of the same NPCs, viewed through objective O2. Scale bar 500 nm. The arrow denotes the optical axis.

References

1. Dempsey, G. T., Vaughan, J. C., Chen, K. H., Bates, M. & Zhuang, X. Evaluation of fluorophores for optimal performance in localization-based super-resolution imaging. *Nat. Methods* **8**, 1027–1036 (2011).

2. Boening, D., Gauthier-Kemper, A., Gmeiner, B. & Klingauf, J. Cluster Recognition by Delaunay Triangulation of Synaptic Proteins in 3D. *Adv. Biosyst.* **1** (2017).

3. Rasband, W. S. *ImageJ*. National Institutes of Health, Bethesda, Maryland, USA (1997-2016). URL https://imagej.nih.gov/ij/.

4. Weber, K., Rathke, P. C. & Osborn, M. Cytoplasmic microtubular images in glutaraldehyde-fixed tissue culture cells by electron microscopy and by immunofluorescence microscopy. *Proc. Natl. Acad. Sci. U.S.A.* **75**, 1820–1824 (1978).

5. Thermo Fisher Scientific Inc. Product data sheet: Goat anti-Mouse IgG (H+L) Highly Cross-Adsorbed Secondary Antibody, Alexa Fluor 647 (2018). URL https://www.thermofisher.com/antibody/product/Goat-anti-Mouse-IgG-H-L-Highly-Cross-Adsorbed-Secondary-Antibody-Polyclonal/A-21236.

6. Beck, M. *et al.* Nuclear pore complex structure and dynamics revealed by cryoelectron tomography. *Sci.* **306**, 1387–1390 (2004).

7. von Appen, A. & Beck, M. Structure determination of the nuclear pore complex with three-dimensional cryo electron microscopy. *J. molecular biology* **428**, 2001–2010 (2016).
